# Gender Differences in the Digital Divide, Digital Back-Feeding, and Health-Related Quality of Life Among Rural Older Adults: Cross-Sectional Study

**DOI:** 10.2196/75925

**Published:** 2025-10-02

**Authors:** Xin Che, Shujun Chai, Dan Zhao, Shirong Chen, Chengchao Zhou

**Affiliations:** 1 Department of Social Medicine and Health Management School of Public Health, Cheeloo College of Medicine Shandong University Jinan China; 2 NHC Key Lab of Health Economics and Policy Research Shandong University Jinan China; 3 Center for Health Management and Policy Research Shandong University Jinan China; 4 Institute of Health and Elderly Care Shandong University Jinan China; 5 School of Population and Health Renmin University of China Beijing China; 6 Advanced Medical Research Institute Shandong University Jinan China

**Keywords:** digital divide, digital back-feeding, health-related quality of life, HRQOL, gender difference

## Abstract

**Background:**

The digital divide has loomed as a global public issue in recent years. However, evidence is limited regarding whether the digital divide is associated with health-related quality of life (HRQOL) and whether digital back-feeding would buffer this association.

**Objective:**

This study aims to explore the role of digital back-feeding in the relationship between the digital divide and HRQOL among older men and women living in rural China.

**Methods:**

We used data from wave 3 of the Shandong Rural Elderly Health Cohort, conducted in 2022. A total of 3242 (n=1946, 60.02% women) rural older adults were included in the analysis. Moderating effect analysis was performed using Tobit regression models and margins plots.

**Results:**

A total of 71.01% (2302/3242) of the participants reported experiencing digital divide. Participants experiencing digital divide were significantly associated with lower HRQOL as measured by EQ-5D-5L scores (β=–0.020; *P*<.001). We found that digital back-feeding buffered the relationship between digital divide and HRQOL (β=0.024; *P*=.02). Furthermore, gender-stratified analyses revealed divergent moderation patterns; a significant buffering role was observed in women (β=0.031; *P*=.02), whereas no substantially significant moderating role emerged in men.

**Conclusions:**

Our study established a significant inverse association between the digital divide and HRQOL among rural adults. Digital back-feeding emerged as a measurable protective buffer mitigating this adverse relationship. Furthermore, this buffering effect was only observed among older women. Policy implications underscore the necessity of gender-tailored digital inclusion strategies, particularly advocating for technology-proficient adult offsprings to prioritize digital engagement with their mothers in digitally marginalized rural communities.

## Introduction

### Background

Population aging has become a global issue of wide concern. As one of the countries in the world experiencing the fastest population aging, China had approximately 280 million people aged more than 60 years in 2022, accounting for 19.8% of its total population [1]. This number is expected to rise to 509 million, accounting for 38.81% of its total population by 2050 [2]. As the population ages and life expectancy increases, improving the health-related quality of life (HRQOL) of older adults has attracted attention from academic and social fields. HRQOL is a multidimensional concept that includes physical functioning, mental health, and socially related roles as perceived by an individual over time [3]. It has been increasingly used as a comprehensive health indicator in health surveys, as poor HRQOL is associated with various adverse outcomes, including loss of functional independence, mental health deterioration, and mortality [4,5]. To promote HRQOL among older people, it is important to identify factors associated with this key health-related outcome.

Digital access is emerging as a social determinant of health. Research has demonstrated that digital technologies directly impact specific aspects of HRQOL in older adults through multiple pathways. For instance, telehealth access has reduced health care barriers through remote monitoring and internet-based consultations [6]. Digital platforms have combated social isolation through communication tools [7]. Furthermore, online health information seeking has empowered informed health care decision-making [8]. People with better access to digital technologies tend to have improved mental health, physical health, and medical decision-making skills [9-12]. Existing literature has predominantly examined quality of life (QOL) in older populations [13-15], and these findings hold critical implications for HRQOL. However, the disparity in different groups’ access to and use of information and communications technologies (ICTs) and the internet often leads to inequalities known as the *digital divide* [16,17]. People affected by the digital divide may miss out on many of the described benefits, causing inequities and disparities in health [18,19], thus reducing QOL. Previous studies have shown that individuals aged more than 60 years account for 41.9% of the total number of nonnetizens [20], and the rate of digital divide could reach up to 90.4% [21] in China. Notably, due to the unequal economic development and resource allocation, the digital divide seems to pose an even greater challenge for older individuals residing in rural areas [18,21,22]. However, the association between the digital divide and HRQOL among rural older adults remains unclear. Understanding the digital challenges faced by rural older adults is crucial for developing strategies to bridge the digital divide and improve their health outcomes.

Digital back-feeding, a new form of postfigurative culture, refers to the teaching behaviors of the younger generation toward the older generation on digital access, use, and literacy [23-25]. Unlike other forms of intergenerational support, such as emotional encouragement or financial aid, digital back-feeding is distinct in its targeted focus on transferring digital skills and knowledge, enabling older adults to navigate technology independently [22,26]. Family members, especially the adult offsprings, are usually considered warm experts who participate in the daily lives of older adults and assist them in solving problems encountered while using technology [27]. While digital support could theoretically come from various family members, including grandchildren or other relatives, adult offsprings typically emerge as the primary source of such assistance in rural areas [28]. This pattern reflects cultural norms, such as filial piety, closer geographic proximity [29], and a more favorable age gap that facilitates effective intergenerational communication and learning [30].

Digital back-feeding is crucial in addressing the digital divide and its impact on HRQOL, as it may bridge generational gaps in digital access and empower older adults to mitigate the negative effects of digital exclusion. Previous studies showed that intergenerational interaction and support can promote internet use and digital literacy among older adults [31,32]. Receiving digital back-feeding could facilitate the integration of older adults into the digital era, foster harmonious parent-child relationships, bolster self-efficacy, and improve subjective well-being [33-37]. A previous study demonstrated that contact with family plays a significant role in the relationship between IT use and psychological well-being [38]. Khalaila and Vitman-Schorr [14] found that social capital, such as being accompanied by family, moderates the positive association between internet use and QOL. With regard to our hypotheses, digital back-feeding is posited as a key moderator that strengthens the relationship between digital literacy and improved HRQOL by facilitating informed health decisions and reducing social isolation. However, the existing studies on digital back-feeding have mainly focused on qualitative research, and the role of digital back-feeding as a moderator between the digital divide and HRQOL has not been convincingly demonstrated.

In theory, the buffering effect model of social support posits that social support enables individuals to alleviate the detrimental effects of stressful events on their physical and mental well-being, thereby improving their overall health [39]. Specifically, while social support does not modify the stressful event itself, it can adjust individuals’ perceptions of the stressors and their own circumstances, thus providing a protective effect. Simultaneously, being profoundly influenced by traditional Chinese culture, support from the younger generation is perceived as a filial duty. Various forms of intergenerational assistance from juniors are seen as forms of repayment to older adults, thereby enhancing the subjective well-being of older adults [40]. Digital back-feeding, which was considered the most crucial means to deal with the digital divide [41], may mitigate the effects of the digital divide on HRQOL.

Another issue deserving more attention is whether the moderating mechanism of the digital divide and HRQOL would differ by gender. Compelling evidence from previous research shows that there is a gender disparity in both access to and use of ICTs, with women consistently lagging behind men [42-44]. Nevertheless, women are commonly perceived as more relationship-oriented; therefore, they may derive greater benefits from social participation compared to men [45,46]. The digital back-feeding within familial contexts also reveals a discernible gender disparity [47]. Specifically, older women demonstrate a more pronounced breadth and depth of digital back-feeding in comparison to men. These disparities may stem from and lead to differential effects of digital back-feeding on HRQOL through differences in social roles, support-seeking behaviors, and health management patterns. For instance, existing literature indicated that women often show greater embeddedness in family networks [48], engage more actively in help-seeking within families [49], and participate more proactively in health information seeking [50,51]. Given this, we speculate that the moderating effect of digital back-feeding between the digital divide and HRQOL among rural older adults might differ by gender.

### Objectives

The aims of this study were as follows: (1) to determine the prevalence of digital divide among older adults in rural China, (2) to explore the association between digital divide and HRQOL, and (3) to examine the moderating role of digital back-feeding in the digital divide–HRQOL relationship and gender difference in this moderating mechanism. This study provides a new perspective to help rural older adults bridge the digital divide.

## Methods

### Data Collection

The data used in this study were obtained from wave 3 of the Shandong Rural Elderly Health Cohort, which was designed to investigate the health status of the older population aged 60 years or more in rural areas of Shandong province. A multistage stratified random sampling method was used to select participants. Details of the study methodology are described in detail elsewhere [52,53]. The baseline survey was completed in June 2019, and follow-up surveys were conducted in 2020 and 2022. In the third wave, 3468 questionnaires were distributed, and 3242 valid questionnaires with complete information were included in this analysis.

### Measures

#### HRQOL Measure

The HRQOL in this study was measured by the EQ-5D-5L questionnaire, which consists of 5 dimensions, including mobility, self-care, usual activities, pain or discomfort, and anxiety or depression, each of which has 5 levels of response (1=no problems, 2=slight problems, 3=moderate problems, 4=severe problems, and 5=extreme problems). The EQ-5D-5L utility values are generated by weighting each dimension of HRQOL using the time trade-off model set for the Chinese general population [54], ranging from –0.391 to 1.000. Higher scores indicate better health.

#### Digital Divide

The participants were asked two questions [55,56]: (1) how frequently they use the internet (including smartphones and computers), and the responses ranged from 1(“never”) to 5 (“almost every day”) and (2) whether they have a smartphone with internet access. If the answer to the former question was “never” or “less than 1 month” and the answer to the latter question was “no,” the rural older adults were classified as “1” denoting digital divide; otherwise, they were classified as “0” denoting no digital divide. This measurement approach captured the “first-level digital divide” by focusing on basic access to digital technologies, which has been widely used in digital divide research among the older adult population [18,57]. Given that basic internet access remains a primary barrier for rural older adults in China [21], this binary classification was selected for its simplicity and practical applicability. Moreover, digital literacy–based measurements tend to underperform in this population due to their limited baseline digital skills and experience [58].

#### Digital Back-Feeding

Whether the older adults received digital back-feeding was a dichotomous variable represented by the question “Whether your child helps you when you encounter difficulties in using digital devices?” If the answer was yes, the participants were classified as “1,” denoting they received digital back-feeding.” If the answer was no, they were classified as “0,”denoting they did not receive digital back-feeding.”

#### Covariate Variable

Covariate variables included sociodemographic characteristics, life behaviors, and health status. Sociodemographic characteristics were measured by gender, age, education, marital status, and household income. Educational attainment was classified into 3 categories: illiterate or elementary school, junior high school, and high school or above. Marital status was divided into 2 categories: single and married, of which the single category included those who were unmarried, divorced, or widowed. Household income was classified into 4 categories: quartile 1, quartile 2, quartile 3, and quartile 4 according to the quartile methods, and the higher quartile represented a higher family economic status. Life behaviors included current smoking status, current drinking status, and performing physical exercise. Health status was measured using the number of chronic diseases experienced. Chronic disease status was divided into 3 categories: 0, 1, and 2 or more.

### Statistical Analysis

We used Stata/MP (version 17.0; Stata Corp) to examine the data and conducted descriptive statistics and correlation analysis. Descriptive statistics were provided to analyze older adults’ characteristics. Continuous variables were summarized using the means and SDs. Categorical variables were reported as frequencies and percentages. Independent samples 2-tailed *t* tests and 1-way ANOVA were used to test for differences in HRQOL between groups. Tobit regression models were used to examine the association between the digital divide and HRQOL because the distribution of the EQ-5D utility index was censored at 1. Compared to ordinary least squares regression, tobit models provided more accurate parameter estimates when dealing with bounded outcomes [59,60]. We evaluated several progressive models. Model 1 of the regression analysis included digital divide and digital back-feeding as predictors of HRQOL, and model 2 was examined to explore whether digital back-feeding is a potential moderator by adding the interaction term (digital divide×digital back-feeding). The interaction term tested whether the slope of the association between digital divide and HRQOL differed across levels of digital back-feeding. A significant interaction suggested that the strength or direction of the relationship between digital divide and HRQOL varied depending on participants’ level of digital back-feeding. All confounders were included to adjust the model. Regarding gender differences in the moderating mechanism, we performed multiple group analyses by fitting the models separately by gender (models 3-6). In addition, the margins plot was used to illustrate the prediction of HRQOL by digital divide and digital back-feeding. The reported CIs were calculated at the 95% level, and *P* values less than.05 were considered statistically significant.

### Ethical Considerations

This study was approved by the ethics committee of Shandong University (20181228). All participants signed informed consent before the survey. The manuscript contains no identifiable features of research participants. All participants received modest household commodities (eg, laundry detergent and towels) valued at approximately $3 USD per session as compensation for their time and participation.

## Results

### Descriptive Statistics

This study included 3242 older people. Among them, 2302 (71.01%) rural older adults experienced digital divide, and the prevalence was higher among older women (1407/1946, 72.3%). Rural older adults who were younger, men, married, had higher educational levels, and did not have chronic diseases reported higher EQ-5D scores. More information on the participants’ characteristics for the whole sample and gender subsamples is presented in Table 1.

**Table 1:** Description and univariate analysis of EQ-5D for all participants, men, and women (N=3242).

Variables		All		Men (n=1296)		Women (n=1946)	
		Participants, n (%)	EQ-5D, mean (SD)	Participants, n (%)	EQ-5D, mean (SD)	Participants, n (%)	EQ-5D, mean (SD)
Total (N=3242)		3242 (100)	0.896 (0.165)	1296 (39.98)	0.905 (0.167)	1946 (60.02)	0.891 (0.163)
**Digital divide**							
	No	940 (28.99)	0.928 (0.112)^a^	401 (30.94)	0.942 (0.111)^a^	539 (27.7)	0.918 (0.112)^a^
	Yes	2302 (71.01)	0.883 (0.180)	895 (69.06)	0.888 (0.185)	1407 (72.3)	0.881 (0.177)
**Digital back-feeding**							
	No	1132 (34.92)	0.894 (0.156)	474 (36.57)	0.905 (0.157)	658 (33.81)	0.886 (0.155)
	Yes	2110 (65.08)	0.898 (0.169)	822 (63.43)	0.904 (0.173)	1288 (66.19)	0.893 (0.167)
**Age (y)**							
	60-69	1098 (33.87)	0.923 (0.130)^a^	414 (31.94)	0.933 (0.134)^a^	684 (35.15）	0.917 (0.127)^a^
	70-79	1762 (54.35)	0.894 (0.165)	720 (55.56)	0.904 (0.166)	1042 (53.55)	0.886 (0.164)
	≥80	382 (11.78)	0.833 (0.222)	162 (12.5)	0.834 (0.219)	220 (11.31)	0.831 (0.225)
**Marital status**							
	Single^b^	744 (22.95)	0.880 (0.186)^c^	181 (13.97)	0.883 (0.193)	563 (28.93)	0.879 (0.184)^d^
	Married	2498 (77.05)	0.901 (0.157)	1115 (86.03)	0.908 (0.162)	1383 (71.07)	0.896 (0.153)
**Education**							
	Illiterate or elementary school	2542 (78.41)	0.888 (0.172)^a^	845 (65.2)	0.891 (0.181)^a^	1697 (87.2)	0.887 (0.167)^a^
	Junior high school	517 (15.95)	0.927 (0.124)	330 (25.46)	0.931 (0.133)	187 (9.61)	0.920 (0.106)
	High school or above	183 (5.64)	0.922 (0.144)	121 (9.34)	0.930 (0.134)	62 (3.19)	0.906 (0.161)
**Chronic diseases, n**							
	0	489 (15.08)	0.961 (0.083)^a^	254 (19.6)	0.964 (0.088)^a^	235 (12.08)	0.959 (0.077)^a^
	1	858 (26.47)	0.919 (0.138)	369 (28.47)	0.935 (0.118)	489 (25.13)	0.907 (0.150)
	≥2	1895 (58.45)	0.869 (0.184)	673 (51.93)	0.866 (0.200)	1222 (62.8)	0.871 (0.175)
**Household income** ^e^							
	Quartile 1	820 (25.29)	0.880 (0.179)^a^	287 (22.15)	0.874 (0.192)^a^	533 (27.39)	0.884 (0.171)^a^
	Quartile 2	801 (24.71)	0.890 (0.169)	351 (27.08)	0.898 (0.167)	450 (23.12)	0.884 (0.171)
	Quartile 3	811 (25.02)	0.902 (0.168)	331 (25.54)	0.918 (0.167)	480 (24.67)	0.891 (0.168)
	Quartile 4	810 (24.98)	0.913 (0.138)	327 (25.23)	0.926 (0.138)	483 (24.82)	0.905 (0.137)
**Current smoking status**							
	No	2562 (79.03)	0.892 (0.168)^c^	776 (59.88)	0.901 (0.177)	1786 (91.78)	0.888 (0.165)^d^
	Yes	680 (20.97)	0.912 (0.148)	520 (40.12)	0.910 (0.151)	160 (8.22)	0.918 (0.137)
**Current drinking status**							
	No	2414 (74.46)	0.887 (0.175)^a^	642 (49.54)	0.883 (0.200)^a^	1772 (91.06)	0.889 (0.164)^d^
	Yes	828 (25.54)	0.924 (0.127)	654 (50.46)	0.926 (0.123)	174 (8.94)	0.914 (0.142)
**Physical exercise**							
	No	1366 (42.13)	0.860 (0.199)^a^	569 (43.9)	0.871 (0.203)^a^	797 (40.96)	0.852 (0.197)^a^
	Yes	1876 (57.87)	0.923 (0.127)	727 (56.1)	0.931 (0.127)	1149 (59.04)	0.918 (0.127)

^a^*P*<.001.

^b^Singles included those who were unmarried (n=28, 0.86%), divorced (n=3, 0.09%), and widowed (n=713, 21.99%) among the whole sample.

^c^*P*<.01.

^d^*P*<.05.

^e^Quartile 1 refers to the lowest income, and quartile 4 refers to the highest income.

### Association Between the Digital Divide and HRQOL

As shown in Table 2, older adults experiencing digital divide were associated with poorer EQ-5D scores compared to those not experiencing digital divide (β=–0.020; *P*<.001), when control variables were included among the whole sample in model 1. Similar associations were observed in the grouped regression (models 3 and 5).

**Table 2:** Standardized coefficients of explanatory variables on health-related quality of life among all participants, men, and women (N=3242).

Exploratory variables			All				Men (n=1296)				Women (n=1946)		
			Model 1^a^, β (SE)		Model 2^b^, β (SE)		Model 3^c^, β (SE)		Model 4^d^, β (SE)		Model 5^c^, β (SE)		Model 6^d^, β (SE)
**Digital divide (reference: no)**													
	Yes	–0.02 (0.005)^e^		–0.036 (0.008)^e^		–0.025 (0.009)^f^		–0.032 (0.012)^f^		–0.017 (0.007)^g^		–0.038 (0.01)^e^	
**Digital back-feeding (reference: no)**													
	Yes	0.001 (0.006)		–0.016 (0.007)^g^		–0.008 (0.009)		–0.016 (0.011)		0.007 (0.007)		–0.016 (0.009)	
	Digital divide × digital back-feeding	—^h^		0.024 (0.01)^g^		—		0.011 (0.016)		—		0.031 (0.013)^g^	
**Gender (reference: men)**													
	Women	0.008 (0.008)		0.008 (0.008)		—		—		—		—	
**Age (y; reference: 60-70 y)**													
	70-80	–0.011 (0.006)		–0.011 (0.006)		–0.002 (0.009)		–0.002 (0.009)		–0.018 (0.007)^g^		–0.018 (0.007)^g^	
	˃80	–0.073 (0.012)^e^		–0.073 (0.012)^e^		–0.065 (0.018)^e^		–0.065 (0.018)^e^		–0.077 (0.016)^e^		–0.077 (0.016)^e^	
**Marital status (reference: single** ^i^ **)**													
	Married	0.006 (0.007)		0.007 (0.007)		0.014 (0.014)		0.014 (0.014)		0.005 (0.009)		0.005 (0.009)	
**Education** **(reference: illiterate or elementary school)**													
	Junior high school	0.023 (0.007)^e^		0.023 (0.007)^e^		0.022 (0.01)^g^		0.022 (0.01)^g^		0.025 (0.009)^f^		0.025 (0.009)^f^	
	High school or above	0.006 (0.011)		0.007 (0.011)		0.014 (0.013)		0.014 (0.013)		–0.012 (0.018)		–0.011 (0.018)	
**Chronic diseases (n; reference: 0)**													
	1	–0.04 (0.006)^e^		–0.04 (0.006)^e^		–0.031 (0.008)^e^		–0.03 (0.008)^e^		–0.047 (0.008)^e^		–0.047 (0.008)^e^	
	≥2	–0.085 (0.006)^e^		–0.085 (0.006)^e^		–0.094 (0.009)^e^		–0.094 (0.009)^e^		–0.081 (0.007)^e^		–0.08 (0.007)^e^	
**Household income** ^j^ **(reference: quartile 1)**													
	Quartile 2	–0.002 (0.009)		–0.002 (0.009)		0.017 (0.014)		0.018 (0.014)		–0.014 (0.011)		–0.014 (0.011)	
	Quartile 3	0.005 (0.009)		0.005 (0.009)		0.027 (0.014)		0.027 (0.014)		–0.009 (0.012)		–0.01 (0.011)	
	Quartile 4	0.01 (0.008)		0.01 (0.008)		0.029 (0.013)^g^		0.028 (0.013)^g^		–0.001 (0.01)		–0.002 (0.01)	
**Smoke (reference: no)**													
	Yes	0.002 (0.007)		0.002 (0.007)		–0.004 (0.009)		–0.004 (0.009)		0.015 (0.011)		0.015 (0.012)	
**Drinking (reference: no)**													
	Yes	0.025 (0.007)^e^		0.025 (0.007)^e^		0.031 (0.008)^e^		0.031 (0.008)^e^		0.011 (0.011)		0.01 (0.011)	
**Physical exercise (reference: no)**													
	Yes	0.062 (0.006)^e^		0.062 (0.006)^e^		0.061 (0.009)^e^		0.061 (0.009)^e^		0.064 (0.008)^e^		0.063 (0.008)^e^	
	Constant	0.925 (0.015)^e^		0.936 (0.015)^e^		0.908 (0.024)^e^		0.913 (0.023)^e^		0.94 (0.015)^e^		0.956 (0.015)^e^	

^a^Model 1 adjusted all cofounders for the whole sample.

^b^Model 2 adjusted all cofounders and the interaction term of digital divide and digital back-feeding for the whole sample.

^c^Models 3 and 5 adjusted all cofounders for the gender subsamples.

^d^Models 4 and 6 adjusted all cofounders and the interaction term of digital divide and digital back-feeding for the gender subsamples.

^e^*P*<.001.

^f^*P*<.01.

^g^*P*<.05.

^h^Data not available.

^i^Singles included those who were unmarried (n=28, 0.86%), divorced (n=3, 0.09%), and widowed (n=713, 21.99%) among the whole sample.

^j^Quartile 1 refers to the lowest income, and quartile 4 refers to the highest income.

### Moderating Role of Digital Back-Feeding Between the Digital Divide and HRQOL

**Figure 1:**
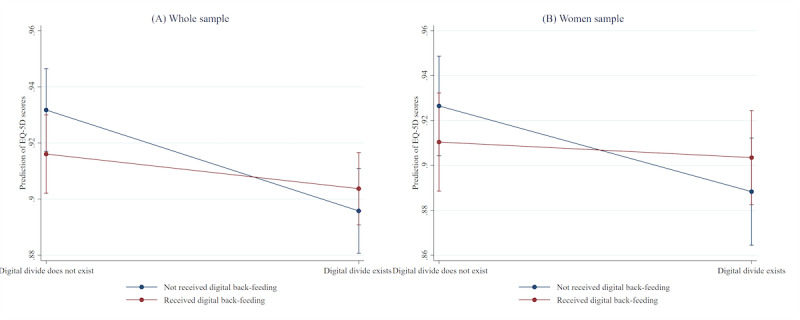
Interaction between digital divide and digital back-feeding in the prediction of EQ-5D scores among the whole and women samples.

Model 2 in Table 2 included the interaction term (digital divide × digital back-feeding) to explore whether intergenerational digital support played a moderating role in the relationship between digital divide and HRQOL. The significant interaction term (β=0.024; *P*=.02) suggested that digital back-feeding was associated with a weaker negative relationship between the digital divide and HRQOL. In the men subsample, no significant moderating effect was observed in the relationship between the digital divide and HRQOL. However, within the women subsample, a significant moderating effect was observed (β=0.031; *P*=.02). Figure 1 illustrates the moderating pattern of digital back-feeding on the association between the digital divide and HRQOL among the whole sample and women subsample.

## Discussion

### Principal Findings

This study first explored the association between the digital divide and HRQOL as well as the role of digital back-feeding as a moderator in this relationship among rural older adults. Our results revealed that the digital divide was associated with poorer HRQOL, and digital back-feeding alleviated this negative association. Moreover, the buffer effect of digital back-feeding between the digital divide and HRQOL was observed in older women participants but not in older men participants. The findings were beneficial to facilitate the development of targeted interventions for individuals to improve HRQOL in rural older adults.

In this study, 71.01% (2302/3242) of the participants experienced digital divide. Although the internet gradually penetrates the middle and older age groups over time [61,62], it remains lower in China and some other low- and middle-income countries [56]. The possible reason was that the advantages of the internet were constrained due to the concurrent occurrence of economic growth and population aging. Older adults face numerous physical or cognitive challenges when learning and using the internet [63]. Furthermore, decreased familiarity, the lack of digital literacy, issues of trust, and concerns about privacy restrict the use of the internet among older adults [64]. Compared with urban areas, rural areas lag in terms of internet infrastructure, education levels, and digital literacy [65]. Thus, as the primary population experiencing “information poverty,” rural older adults face barriers to enjoying the dividends brought by the development of digital technology and require more assistance.

Results from our study indicated that the digital divide was negatively associated with HRQOL among Chinese rural older adults, which is consistent with previous studies [13,14]. The possible explanation is that the internet enables older adults to access more health-related information and medical services [66,67]. Rural older adults with poor internet accessibility are blocked from accessing health knowledge, which affects their health-related behaviors affected, deteriorates their physical and mental health, and ultimately reduces HRQOL. Moreover, the internet provides older adults with additional options for social participation and access to information related to their personal interests. Rural older people who encounter the digital divide may feel socially isolated and have reduced social interaction, resulting in feelings of loneliness, unhappiness, and boredom [68]. These findings imply that promoting internet use to bridge the digital divide could be beneficial for older people’s HRQOL, leading to better health outcomes.

Our study also revealed that digital back-feeding moderated the association between digital divide and HRQOL. Specifically, digital back-feeding buffers the negative impact of digital divide on HRQOL among rural older adults. There are several possible explanations for this finding. First, support with digital technology from their offspring can help older adults better adapt to digital life and build better connections with society [69]. It can strengthen their sense of social belonging, enhance their self-worth, enrich their spiritual life, and be beneficial for their mental health. Second, digital back-feeding can improve health outcomes by increasing digital health literacy among older adults. Previous studies found that better digital health literacy is associated with positive health behaviors and better HRQOL [11,70], while digital back-feeding can improve parents’ digital literacy [71]. Third, family constitutes the pivotal domain for digital feedback [72]. For rural older adults, households function as the fundamental units for health promotion and significantly contribute to their overall well-being. Family support and intergenerational interactions help mitigate feelings of loneliness and reduce the risk of psychological diseases by fostering stronger bonds across different age groups [37,38,73]. These supportive relationships ultimately enhance the HRQOL of older adults. Thus, digital back-feeding could mitigate the possibility of rural older adults experiencing the digital divide having worse HRQOL.

This study examined gender differences in the moderation mechanism. Digital back-feeding buffered the negative correlation between the digital divide and HRQOL for older women after adjusting for other confounding factors but not among men. Consistent with previous studies, women are less likely to access and use ICTs than men [42,43,74]. This disparity can be somewhat elucidated through a life course perspective [75]. Men and women have encountered distinct opportunities over the years, especially in the realms of educational attainment, paid employment, social concepts, and caregiving responsibilities, which have subsequently influenced their exposure to ICTs. Therefore, digital back-feeding activities aimed at women are more likely to show positive impacts. Compared to men, women’s social worlds were more confined to their locality, with the domestic and emotional labor of “making” a home being central to their identity [76]. This distribution of duties tends to increase their reliance on family members for support, particularly when confronted with new technologies. Conversely, men may participate less in these intrafamilial support activities and thus benefit less from digital support from their offsprings. Another explanation was that older women are more likely to articulate and share their emotions and demonstrate superior effectiveness in establishing informal networks [77]. These gender-specific findings have important implications for targeted interventions to bridge the digital divide among rural older adults. Given that women showed greater responsiveness to digital back-feeding, family-integrated digital literacy programs should be prioritized. Adult offsprings can serve as digital mentors in these programs, providing collaborative support that matches women’s relational orientations. For men, digital back-feeding demonstrated limited benefits due to socialized independence; therefore, interventions should emphasize autonomous learning approaches. Peer-led training or individual tutorials focusing on practical applications may be more acceptable while building self-efficacy.

The findings of this study provide new inspiration for rural older adults to enhance HRQOL, especially highlighting the need for gender-tailored interventions. It is crucial to mobilize social support from both formal and informal organizations. First, policymakers should safeguard digital rights for older adults by strengthening the retrofitting of digital platforms for aging and enhancing digital health literacy. For example, local governments can establish digital literacy training in community centers, offering family-oriented sessions where adult offsprings guide older women in using smartphones for health management and communication. Second, rural communities should promote digital development by improving internet infrastructure and collaborating with telecommunication providers to offer affordable smartphones and internet plans, ensuring that all older adults can easily access online services. Third, adult offsprings should be encouraged to spend more time and patience assisting older adults, especially older women, to use digital technologies for communication or entertainment. Last but not least, social groups and organizations should actively promote digital inclusion by providing volunteer support and technical assistance, while internet service providers should develop age-friendly interfaces to reduce access barriers. By implementing these targeted and practical measures, rural areas can more effectively bridge the digital divide and improve the health and QOL of all older adults, with particular attention to the unique needs of women.

### Limitations

Despite the contributions made to the existing literature, there are several limitations in this study. First, this study used cross-sectional data, which may introduce recall bias. In the future, longitudinal designs will be used to verify this relationship. Second, our measurement approaches have inherent constraints. The digital divide was assessed primarily through basic access metrics rather than comprehensive digital literacy or quality of internet use, which may have overlooked nuanced variations in digital engagement affecting HRQOL. This limitation would be remedied in the future study. Third, the findings’ generalizability should be interpreted cautiously, as data were self-reported and drawn exclusively from Shandong Province, China. Broader, multiregional studies with objective measures are recommended to validate and extend these results.

### Conclusions

In conclusion, 71.01% (2302/3242) of the participants reported having experienced the digital divide. Our findings demonstrated that the digital divide was negatively associated with HRQOL, and digital back-feeding moderated this relationship among rural older adults. Moreover, the buffering effect was observed in older women but not in older men. Recognizing the relationship between the digital divide and HRQOL provides insights for bridging the digital divide and enhancing HRQOL among older adults. This study suggests that broad measures should be implemented, and efforts should be taken to help the rural older population bridge the digital divide and integrate into the digital society.

## Abbreviations

HRQOL: health-related quality of life
ICT: information and communication technology
QOL: quality of life
